# Banting memorial lecture 2022: ‘Type 2 diabetes and nonalcoholic fatty liver disease: Partners in crime’

**DOI:** 10.1111/dme.14912

**Published:** 2022-07-20

**Authors:** Christopher D. Byrne

**Affiliations:** ^1^ Division of Endocrinology & Metabolism University Hospital Southampton and University of Southampton Southampton UK

**Keywords:** GLP‐1 receptor agonists, insulin resistance, liver fibrosis, nonalcoholic fatty liver disease, pioglitazone, type 2 diabetes

## Abstract

Nonalcoholic fatty liver disease (NAFLD) was first described in the 1980s, but in the 21st century, NAFLD has become a very common condition. The explanation for this relatively recent problem is in large part due to the recent epidemic of obesity and type 2 diabetes (T2DM) increasing the risk of NAFLD. NAFLD is a silent condition that may not become manifest until severe liver damage (fibrosis or cirrhosis) has occurred. Consequently, NAFLD and its complications often remain undiagnosed. Research evidence shows that NAFLD is extremely common and some estimates suggest that it occurs in up to 70% of people with T2DM. In the last 5 years, it has become evident that NAFLD not only increases the risk of cirrhosis, primary liver cancer and end‐stage liver disease, but NAFLD is also an important multisystem disease that has major implications beyond the liver. NAFLD increases the risk of incident T2DM, cardiovascular disease, chronic kidney disease and certain extra‐hepatic cancers, and NAFLD and T2DM form part of a vicious spiral of worsening diseases, where one condition affects the other and vice versa. Diabetes markedly increases the risk of liver fibrosis and liver fibrosis is the most important risk factor for hepatocellular carcinoma. It is now possible to diagnose liver fibrosis with non‐invasive tools and therefore it is important to have clear care pathways for the management of NAFLD in patients with T2DM. This review summarises key recent research that was discussed as part of the Banting lecture at the annual scientific conference in 2022.

## 1 INTRODUCTION

Nonalcoholic fatty liver disease (NAFLD) was first described in 1980,^1^ but in the 21st century, NAFLD has become a very common condition. The explanation for this is in large part due to the epidemic of obesity and type 2 diabetes (T2DM) causing NAFLD. NAFLD represents a spectrum of liver fat‐associated conditions that begin with liver steatosis and progresses to steatohepatitis, liver fibrosis and cirrhosis. With the increasing severity of liver fibrosis, there is also a marked increase in the risk of hepatocellular carcinoma.^2^ NAFLD is a silent condition that may not become manifest until severe liver damage has occurred, and therefore NAFLD and its complications often remain undiagnosed in people with diabetes.

The prevalence of NAFLD increases in patients with T2DM and/or metabolic comorbidities and the metabolic syndrome (MetS), defined by the presence of at least three metabolic alterations amongst elevated waist circumference (≥94 cm in males; ≥80 cm in females in Europids), increased triglycerides (≥1.7 mmol/L or 150 mg/dL), reduced HDL‐C (≤1.0 mmol/L or 40 mg/dL in men; ≤1.3 mmol/L or 50 mg/dL in women), increased blood pressure (systolic pressure ≥ 130 mmHg and/or diastolic pressure ≥ 85 mmHg or antihypertensive drug treatment) and increased fasting glucose (≥5.6 mmol/L or 100 mg/dL or antihyperglycemic treatment).^3^ Many of these features of MetS may be present with NAFLD and the prevalence of NAFLD may be up to 70%–80% in patients with T2DM.^4^, ^5^ It is now estimated that NAFLD affects a quarter of the world's adult population^6^ and a further concern is that the epidemic of obesity, metabolic dysfunction and T2DM in young people^7^ will likely increase the prevalence and complications of NAFLD in the near future.^8^, ^9^

Data from NHANES (1999–2016) and NHANES III (1988–1994) have also been used to investigate national estimates and temporal trends for NAFLD, based on different fibrosis severity.^10^ In this study, NAFLD was determined by ultrasound showing moderate to severe steatosis. For those without ultrasound, NAFLD was determined by the US‐Fatty Liver Index score of ≥30. Hepatic fibrosis was assessed using the FIB‐4 score. Annual per cent change (APC) was calculated using the join‐point regression model. Ten thousand eight hundred fifty‐four individuals were included (mean age 43.5 years; 47.5% male; 75.7% non‐Hispanic white) and 37.7% had NAFLD. Amongst these subjects, based on the FIB‐4 score, 80% had low‐risk, 18.6% had moderate‐risk and 1.4% had high‐risk NAFLD. Subjects with NAFLD and moderate/high‐risk fibrosis (compared with low‐risk), were more likely to have hypertension, hyperlipidemia, diabetes, cardiovascular disease and MetS. NAFLD prevalence increased from 29.5% in 1999–2000 to 40.3% in 2015–2016 (APC: 2.78%, p < 0.02); moderate‐risk NAFLD increased from 6.26% to 14.17% (APC: 5.34%, p < 0.02) and high‐risk NAFLD increased from 0.49% to 1.15% (APC 9.72%, p < 0.02). Thus, these important data provide clear evidence that there is a secular trend in a given population and the prevalence of NAFLD is clearly increasing over time. This review summarises key recent research linking T2DM and NAFLD that was discussed as part of the Banting lecture at the Diabetes UK annual scientific conference in the Spring of 2022. The review illustrates how T2DM and NAFLD form part of a ‘vicious spiral of worsening morbidity’ with NAFLD adversely influencing diabetes‐related morbidity and T2DM influencing the severity of the liver disease. The review also discusses key research over the last decade that had focussed on NAFLD as a multisystem disease: A liver condition that increases the risk of many important extrahepatic diseases.

## 2 HOW SHOULD NAFLD BE DIAGNOSED AND MONITORED?

Liver biopsy and the assessment of liver histology are recognised as the gold standard for the assessment of liver disease severity in NAFLD. However, the use of this ‘gold’ standard staging of liver disease severity is recognised to be impractical, costly, risky and not feasible for monitoring treatment responses in routine clinical practice. The staging of liver disease with NAFLD involves the severity of different criteria (steatosis, inflammation and fibrosis)^11^, ^12^, ^13^ and commonly the severity of liver fibrosis severity is assessed according to four categories from zero (F0) to cirrhosis (F4). The diagnosis of NAFLD requires the exclusion of both secondary causes and alcohol consumption ≥30 g per day for men and ≥ 20 g per day for women.^14^ Recently, a consensus of experts proposed overcoming problems with the current nomenclature ‘NAFLD’ by adopting a more ‘positive’ definition in the acronym MAFLD, referring to Metabolic dysfunction‐Associated Fatty Liver Disease.^15^ This new classification and characterisation of fatty liver disease employs metabolic dysfunction as a focus and utilises diagnostic criteria that are independent of the presence of other causes of chronic liver disease. MAFLD also allows for modest alcohol consumption that is potentially hazardous and above the thresholds allowed to diagnose NAFLD. MAFLD also allows for the presence of co‐existing other chronic liver diseases.

The presence of steatosis can be assessed by use of the ultrasound component in recent ‘Fibroscanners’ (the controlled attenuation parameter [CAP]), and liver fibrosis can be assessed using the pressure wave measurement of liver stiffness, as a proxy measurement of liver fibrosis in the absence of other factors that might increase liver stiffness. Liver fibrosis severity is defined as follows: mild (F1) if LSM ≥7.0–8.1 kPa, moderate fibrosis (F2) if ≥8.2–9.6 kPa, advanced fibrosis (F3) if ≥9.7–13.5 kPa and cirrhosis (F4) if ≥13.6 kPa, and these kPa thresholds have recently been validated in a large key validation study with histological assessment of liver disease severity.^16^ Using Fibroscan to assess both liver steatosis and liver fibrosis in a prospective cohort study of 776 patients with T2DM, 60.3% had severe steatosis, whilst 19.4% had advanced fibrosis.^17^ In this study, female sex, BMI, waist circumference, increased levels of AST, total cholesterol, triglycerides, blood glucose and high LSM were all associated with severe steatosis. BMI, waist circumference, increased levels of AST, HbA1c and CAP were all associated with advanced fibrosis.^17^ Moderate‐to‐advanced fibrosis (F2 or higher) is an established risk factor for cirrhosis and overall mortality, and it has recently been estimated that this level of severity of liver fibrosis affects at least 15% of patients with T2DM.^18^

Patients in high‐risk groups such as those with MetS or T2DM are at higher risk of more severe liver disease (e.g., liver fibrosis, cirrhosis and primary liver cancer) and co‐morbidities associated with NAFLD.^19^ Liver fibrosis and cirrhosis are the most important predictor of mortality in NAFLD and the presence of liver fibrosis, is associated with increased all‐cause, liver‐related and cardiovascular mortality.^10^ A recent systematic review and meta‐analysis involving 1495 NAFLD patients with 17,452 patient years of follow‐up, investigated the association between the severity of liver fibrosis and both liver‐related and all‐cause mortality.^20^ Compared to NAFLD patients with no fibrosis (stage 0), NAFLD patients with fibrosis were at an increased risk for all‐cause mortality and this risk increased with increases in the stage of fibrosis: stage 1, mortality rate ratios (MRR) = 1.58 (95% CI 1.19–2.11); stage 2, MRR = 2.52 (95% CI 1.85–3.42); stage 3, MRR = 3.48 (95% CI 2.51–4.83); stage 4, MRR = 6.40 (95% CI 4.11–9.95). Importantly, the results were more pronounced as the risk of liver‐related mortality increased exponentially with each increase in the stage of fibrosis: stage 1, MRR = 1.41 (95% CI 0.17–11.95); stage 2, MRR = 9.57 (95% CI 1.67–54.93); stage 3, MRR = 16.69 (95% CI 2.92–95.36); stage 4, MRR = 42.30 (95% CI 3.51–510.34).^20^ Importantly, a recent systematic review and meta‐analysis of population‐based cohort studies investigated the association between metabolic risk factors (including T2DM) and the development of advanced liver disease in NAFLD. Databases were searched up to January 2020. T2DM data were obtained from 12 studies, including 22.8 million individuals who were followed up for a median of 10 years (IQR 6.4 to 16.9) and who experienced 72,792 liver events.^21^ These data showed that T2DM was associated with an increased risk of incident severe liver disease events (adjusted HR 2.25, 95% CI 1.83–2.76, p < 0.001). As mentioned above, low HDL‐C and increased fasting triglyceride concentrations and hypertension are features of the MetS, and these features occur frequently with NAFLD. In the above meta‐analysis, these features of MetS were also independently associated with increased development of advanced liver disease in NAFLD.^21^

## 3 NAFLD IS A MULTISYSTEM DISEASE WITH EFFECTS BEYOND THE LIVER

### 3.1 T2DM and NAFLD act as ‘partners in crime’ to increase the risk of extra‐hepatic complications

It is also now clear that NAFLD is a multisystem disease^22^ that requires a multidisciplinary, holistic approach to its management.^23^ NAFLD increases the risk of hepatocellular carcinoma (HCC).^24^ Evidence suggests that NAFLD not only affects the liver but is an independent risk factor for several other diseases, including T2DM,^25^ chronic kidney disease^26^ and non‐hepatic cancers.^27^ Recently, we have investigated effect‐modification by sex and by menopause on the association between NAFLD and T2DM; and we also assessed whether a diagnosis of NAFLD adds to conventional diabetes risk factors for predicting T2DM.^28^ In a large cohort study of ~245,000 subjects without diabetes at baseline, these data showed that NAFLD, including more severe NAFLD, is a stronger risk factor for incident T2DM in premenopausal women than in post‐menopausal women or men, and protection against developing T2DM is lost in pre‐menopausal women with NAFLD. Importantly, these data also showed that the addition of NAFLD to conventional diabetes risk factors, improved risk prediction for incident T2DM in both sexes, with a greater improvement in women than men.^28^

Although a little more controversial, the weight of evidence also now suggests that NAFLD is also a risk factor for cardiovascular and cardiac disease.^29^, ^30^, ^31^ In a recent meta‐analysis, showing that NAFLD was associated with a ~ 50% increase in the risk of developing CVD,^29^ univariable meta‐regression analyses to examine the effect of potential moderator variables, showed there was a significant positive association between the proportion of patients with pre‐existing T2DM (*p* = 0.001) and also mean plasma LDL‐cholesterol concentrations (*p* = 0.041), with the risk of NAFLD‐related CVD events. Thus, it seems likely that there is also a modifying influence of T2DM (and LDL‐cholesterol) to further increase the risk of developing CVD, in patients with NAFLD. The study characteristics of included studies, effect sizes for the increases in risk for each outcome (incident diabetes, incident cardiovascular disease, incident CKD and incident extra‐hepatic cancers), and the interpretation of each of these meta‐analyses are summarised in Table 1.

**TABLE 1:** NAFLD as a multisystem disease: recent meta‐analyses describing the increased risk of incident diabetes, incident cardiovascular disease, incident chronic kidney disease and incident extra‐hepatic cancers with NAFLD

Publication	Study aims	Study characteristics	Summary estimate of risk (e.g., HR (95% CIs) of outcome associated with NAFLD	Comments and interpretation
Mantovani et al.^25^	To ascertain the risk of incident diabetes associated with NAFLD	33 studies (501,022 individuals), 30.8% with NAFLD 27,953 cases of incident diabetes over a median of 5 years (IQR: 4.0–19 years) were included Meta‐analysis was performed using random‐effects modelling	Patients with NAFLD had a higher risk of incident diabetes than those without NAFLD (*n* = 26 studies; random‐effects HR 2.19, 95% CI 1.93 to 2.48; *I* ^*2*^ = 91.2%). Patients with more ‘severe’ NAFLD were also more likely to develop incident diabetes (*n* = 9 studies; random‐effects HR 2.69, 95% CI 2.08 to 3.49; *I* ^*2*^ = 69%). This risk markedly increased across the severity of liver fibrosis (*n* = 5 studies; random‐effects HR 3.42, 95% CI 2.29 to 5.11; *I* ^2^ = 44.6%). All risks were independent of age, sex, adiposity measures and other common metabolic risk factors. Sensitivity analyses did not alter these findings. The funnel plots did not reveal any significant publication bias	PubMed, Scopus and Web of Science databases from January 2000 to June 2020 using predefined keywords to identify observational studies with a follow‐up duration of at least 1 year, in which NAFLD was diagnosed by imaging techniques or biopsy The meta‐analysis shows that NAFLD is associated with a ~ 2.2‐fold increased risk of incident diabetes. This risk parallels the underlying severity of NAFLD The results support those by the authors in an earlier and smaller meta‐analysis in 2018 (Diabetes Care. 2018 Feb;41[2]:372–382)
Mantovani et al.^29^	A meta‐analysis of observational studies to quantify the magnitude of the association between NAFLD and the risk of incident CVD events	36 longitudinal studies with aggregate data on 5,802,226 middle‐aged individuals (mean age 53 years [SD 7]) and 99,668 incident cases of fatal and non‐fatal CVD events over a median follow‐up of 6·5 years (IQR 5·0–10·2) Meta‐analysis was performed using random‐effects models to obtain summary hazard ratios (HRs) with 95% CIs. The quality of the evidence was assessed with the Cochrane risk of bias tool	NAFLD was associated with a moderately increased risk of fatal or non‐fatal CVD events (pooled random‐effects HR 1·45, 95% CI 1·31–1·61; *I* ^2^ = 86·18%). This risk markedly increased across the severity of NAFLD, especially the stage of fibrosis (pooled random‐effects HR 2·50, 95% CI 1·68–3·72; *I* ^2^ = 73·84%). All risks were independent of age, sex, adiposity measures, diabetes, and other common cardiometabolic risk factors Sensitivity analyses did not modify these results	PubMed, Scopus and Web of Science searched from database inception to July 1st 2021 NAFLD was diagnosed by imaging, International Classification of Diseases codes, or liver biopsy The primary outcomes were CVD death, non‐fatal CVD events, or both NAFLD is associated with an increased long‐term risk of fatal or non‐fatal CVD events. CVD risk is further increased with more advanced liver disease, especially with higher fibrosis stage. These results provide evidence that NAFLD might be an independent risk factor for CVD morbidity and mortality N.B. Univariable meta‐regression analyses to examine the effect of potential moderator variables showed a significant positive association between the proportion of patients with pre‐existing type 2 diabetes (*p* = 0·001) or mean plasma LDL‐cholesterol concentrations (*p* = 0·041) and the risk of NAFLD‐related CVD event.
Mantovani et al.^27^	A meta‐analysis of observational studies to quantify the magnitude of the association between non‐alcoholic fatty liver disease (NAFLD) and risk of extrahepatic cancers	10 cohort studies with 182,202 middle‐aged individuals (24.8% with NAFLD) and 8485 incident cases of extrahepatic cancers at different sites over a median follow‐up of 5.8 years Meta‐analysis was performed using random‐effects modelling	NAFLD was significantly associated with a nearly 1.5‐fold to twofold increased risk of developing GI cancers (oesophagus, stomach, pancreas or colorectal cancers). Furthermore, NAFLD was associated with an approximately 1.2‐fold to 1.5‐fold increased risk of developing lung, breast, gynaecological or urinary system cancers. All risks were independent of age, sex, smoking, obesity, diabetes or other potential confounders. The overall heterogeneity for most of the primary pooled analyses was relatively low. Sensitivity analyses did not alter these findings. Funnel plots did not reveal any significant publication bias	PubMed, Scopus and Web of Science databases searched from the inception date to 30 December 2020 using predefined keywords to identify observational cohort studies conducted in individuals, in which NAFLD was diagnosed by imaging techniques or International Classification of Diseases codes. No studies with biopsy‐proven NAFLD were available for the analysis This large meta‐analysis suggests that NAFLD is associated with a moderately increased long‐term risk of developing extrahepatic cancers over a median of nearly 6 years (especially GI cancers, breast cancer and gynaecological cancers). Further research is required to decipher the complex link between NAFLD and cancer development
Mantovani et al.^26^	A meta‐analysis of observational studies to quantify the magnitude of the association between NAFLD and the risk of incident chronic kidney disease (CKD)	13 studies with 1,222,032 individuals (28.1% with NAFLD) and 33,840 cases of incident CKD stage ≥3 (defined as estimated glomerular filtration rate < 60 ml/min/1.73 m^2^, with or without accompanying overt proteinuria) over a median follow‐up of 9.7 years were included Meta‐analysis was performed using random‐effects modelling	NAFLD was associated with a moderately increased risk of incident CKD (*n* = 10 studies; random‐effects HR 1.43, 95% CI 1.33 to 1.54; *I* ^*2*^ = 60.7%). All risks were independent of age, sex, obesity, hypertension, diabetes and other conventional CKD risk factors. Sensitivity analyses did not alter these findings. Funnel plot did not reveal any significant publication bias	PubMed, Web of Science and Scopus were searched from January 2000 to August 2020 using predefined keywords to identify observational studies with a follow‐up duration of ≥1 year, in which NAFLD was diagnosed by blood biomarkers/scores, International Classification of Diseases codes, imaging techniques or biopsy This large and updated meta‐analysis indicates that NAFLD is significantly associated with a ~ 1.45‐fold increased long‐term risk of incident CKD stage ≥3. Further studies are needed to examine the association between the severity of NAFLD and risk of incident CKD

As mentioned above, when metabolic dysfunction, manifest by the presence of co‐existing features of the MetS^3^ or T2DM, occurs with liver fat, the term MAFLD can be it has been used to describe NAFLD.^15^ There is also now recent evidence to suggest that MAFLD is also associated with an increased risk of CVD.^*32*^ Importantly, a bi‐directional association exists between NAFLD and T2DM, with NAFLD increasing the risk of T2DM and T2DM increasing the risk of severe liver disease and specifically increasing the risk of liver fibrosis, cirrhosis and HCC.^23^ In patients with T2DM, the presence of NAFLD also increases the risk of incident or recurrent HCC by ~ 20‐fold.^33^ Therefore, this evidence lends weight to the notion that NAFLD should be identified in patients with T2DM, so that liver fibrosis can be assessed; not least, because liver fibrosis is a key risk factor for cirrhosis and HCC.^34^ Given that T2DM and NAFLD are also independent risk factors for CVD, CKD and obesity‐related cancers, the combination of both NAFLD and type 2 occurring together is likely to have a greater impact on the development of extra‐hepatic complications. Thus, it could be argued that T2DM and NAFLD are ‘partners in crime’ that act together to increase the risk of both hepatic and extra‐hepatic complications.

## 4 NAFLD, T2DM, AND CARDIOVASCULAR AND CARDIAC DISEASE

There are several mechanistic links that might explain why NAFLD increases the risk of CVD. It is beyond the scope of this review to discuss all of those mechanisms (for further discussion of these subjects see).^35^ In people with NAFLD and also T2DM, regardless of the presence of NAFLD, people with T2DM, insulin resistance and MetS often have a form of dyslipidaemia called the atherogenic lipoprotein phenotype.^36^ This discrete dyslipidaemia was proposed as a marker of increased coronary heart disease risk and was first described in 1990 by Melissa Austin and colleagues.^36^ The atherogenic lipoprotein phenotype was associated with increases in plasma levels of triglyceride and apolipoprotein B, with a mass of very low and intermediate‐density lipoproteins, and with decreases in high‐density lipoprotein (HDL) cholesterol, HDL2 mass, and plasma levels of apolipoprotein A‐I.^36^ The liver has a key role in synthesising triglyceride‐rich lipoproteins associated with apolipoprotein B‐100 (atherogenic lipoproteins) that might explain, at least in part, the association between NAFLD and increased risk of CVD.^37^ Hepatic triglyceride metabolism can result in hypertriglyceridaemia, although increased de novo hepatic lipogenesis, mediated by the liver X receptor and sterol regulatory element‐binding protein 1c, can in turn drive liver injury in NAFLD.^38^ VLDL‐triglyceride secretion has a positive correlation with intrahepatic triglyceride content and decreased plasma VLDL clearance results in the accumulation of triglyceride‐rich remnant particles, which can increase the risk of CVD.^38^ Hypertriglyceridaemia also produces dysfunctional HDL in patients with NAFLD and is also associated with deleterious changes in endothelial cell function^39^ that are associated with increased atherosclerotic cardiovascular disease risk. Since total LDL‐C concentration is usually not raised with the atherogenic lipoprotein phenotype, the lipid abnormality is usually dismissed and a patient who is at increased risk of CVD is unfortunately not treated with a statin. It is conceivable that in the future we may have treatments that not only treat liver disease in NAFLD but also treat the atherogenic lipoprotein phenotype. However, in the meantime, it is important to use lipid‐lowering strategies that are known to decrease apolipoprotein B100 concentrations. Such lipid‐lowering agents are statins, ezetimibe and PCSK9 inhibitors. Presently, PCSK9 inhibitors are only available by injection and are expensive, and, therefore, the default position should be to use statins to lower apolipoprotein B100 containing lipoprotein, plus or minus ezetimibe if needed, bearing in mind that both groups of drugs are safe in patients with NAFLD.

Polymorphisms in certain genes predispose individuals to develop more severe liver disease in NAFLD, e.g. patatin‐like phospholipase domain‐containing protein 3 (PNPLA3); trans‐membrane 6 super family 2 (TM6SF2); glucokinase regulator (GCKR); membrane‐bound O‐acyltransferase domain containing 7 (MBOAT7).^40^ In addition, in a recent *large mu*lti‐cohort exome‐wide association study focused on serum ALT levels, a sequence variant of apolipoprotein E (*APOE)* has been also identified that is associated with NAFLD,^41^ and this genetic variant is well known to be associated with higher risks of both Alzheimer's disease^42^ and dyslipidaemia.^43^ *Polymorphisms in PNPLA3 and TM6SF2 (*i.e. *PNPLA3 rs738409 c.444 C > G p.I148M and* TM6SF2 rs58542926 C > T E167K*) may potentially provide further insight into informing us why NAFLD might act to increase risk of CVD and also why there is the heterogeneity of CVD risk in patients with NAFLD. Both PNPLA3 rs738409 c.444 C > G p.I148M and* TM6SF2 rs58542926 C > T E167K polymorphisms are quite common in patients in NAFLD, and these polymorphisms may attenuate CVD risk. Although polymorphisms in both PNPLA3 and TM6SF2 *(*i.e. *PNPLA3 rs738409 c.444 C > G p.I148M and* TM6SF2 rs58542926 C > T E167K*)* are well known to be associated with more severe liver disease, these polymorphisms in both genes act to decrease VLDL levels and thereby potentially protect the vasculature from the normal increase in atherogenic VLDL and the development of the atherogenic lipoprotein phenotype that would normally occur in patients with NAFLD. The effect of these moderator polymorphisms to attenuate the risk of CVD is discussed in more detail in a recent review of the relationship between NAFLD and CVD.^35^

With the burgeoning 21st problem of obesity, NAFLD has become a common disease that is often present but remains often undiagnosed in the adult population. Thus, in large registry studies investigating associations between NAFLD and outcomes such as CVD,^44^ it is not possible to prove that subjects in the control (reference) group, do not have NAFLD. When undiagnosed NAFLD occurs in subjects in the reference group, this causes misclassification bias, and misclassification bias always attenuates the strength of any association between the exposure variable (i.e. NAFLD) and the outcome (CVD), towards the null. Moreover, amongst the few published NAFLD histology cohorts, that have investigated the association between NAFLD and the risk of incident CVD, most have been limited by small sample sizes (e.g., several hundred subjects) with few recorded outcomes and imprecise estimates of risk across NAFLD histological categories. Thus, in cohort studies where histological data were available to gauge liver disease severity, studies were most likely too small, with too few CVD events, to test the strength of any association between the different stages of liver disease severity and CVD events. That said, Simon et al. recently presented important data from a nationwide cohort of Swedish adults with histologically‐confirmed NAFLD and without pre‐existing CVD at baseline (1966–2016; *n* = 10,422).^30^ In this well‐conducted cohort study, the authors investigated the incidence of major adverse cardiovascular events (MACE) (defined as nonfatal ischaemic heart disease, stroke, congestive heart failure or CVD mortality), according to the presence and histological severity of NAFLD. NAFLD was defined from prospectively recorded histopathology, and categorized as simple steatosis (68.5% of the cohort), non‐fibrotic steatohepatitis (NASH, 11.4%), non‐cirrhotic fibrosis (14.9%) and cirrhosis (5.2%), respectively. Patients with NAFLD (*n* = 10,422) were matched to ≤5 population controls without NAFLD or CVD, by age, sex, calendar year and country (*n* = 46,517). Over a median of 13.6 years of follow‐up, incident MACE was confirmed in 2850 NAFLD patients (27.3%) and in 10,648 matched controls (22.9%). After adjustment for common cardiometabolic risk factors and potential confounders, NAFLD was significantly associated with a nearly 65% increased risk of incident MACE. Furthermore, the risk of incident MACE increased monotonically with worsening NAFLD severity (P‐value for trend = 0.02). Specifically, compared to matched controls, the absolute rate differences and corresponding fully‐adjusted hazard ratios (aHR) were significantly increased in patients with both simple steatosis (7.0/1000 person‐year [PY]; aHR = 1.58, 95%CI = 1.50–1.67) and NASH without fibrosis (8.1/1000PY; aHR = 1.52, 95%CI = 1.32–1.75), and they were further amplified in patients with non‐cirrhotic fibrosis (11.1/1000PY; aHR = 1.67, 95%CI = 1.47–1.89), or in those with cirrhosis (27.2/1000PY; aHR = 2.15, 95%CI = 1.77–2.61). Interestingly, and worthy of further study, in stratified analyses, the significant association between NAFLD and incident MACE outcomes appeared stronger amongst women than men, amongst patients diagnosed with NAFLD at younger ages, and also amongst those with a positive family history of premature CVD.

Thus, Simon et al. show convincingly that NAFLD is associated with significant excess risk of individual MACE outcomes, including nonfatal ischaemic heart disease, stroke, CVD mortality and also importantly congestive heart failure. However, the mechanisms by which the increased risk of congestive heart failure occurs remain uncertain. Whether any increased risk of heart failure occurs as a result of ischaemic heart disease induced by, for example, the atherogenic lipoprotein phenotype (described above), or whether the increased risk of heart failure occurs due to cardiac remodelling, or a shared increased risk of fibrosis (occurring in both liver and heart), remains uncertain. In a recent brief narrative review, we have discussed the association between NAFLD and increased risk of new‐onset heart failure.^45^ In that review, we have also discussed the underlying mechanisms that link these two diseases, discussed the associations between NAFLD and cardiac arrthymias such as atrial fibrillation and summarized pharmacological treatments for NAFLD that might also reduce the risk of HF.

Recently, with the continued ongoing debate as to whether NAFLD is an active contributor that increases CVD risk, a two‐sample Mendelian randomization (MR) analysis using summary‐level data to assess the association between genetically predicted NAFLD (i.e., chronically elevated serum alanine aminotransferase levels [cALT], imaging‐based and biopsy‐confirmed NAFLD), and risk of coronary artery disease was undertaken.^46^ Considering the influence of NAFLD‐susceptibility genes *(*i.e., *PNPLA3 rs738409 c.444 C > G p.I148M and* TM6SF2 rs58542926 C > T E167K*)* that also decrease VLDL secretion (i.e., an example of horizontal pleiotrophy), analyses were repeated after exclusion of these NAFLD susceptibility genes that also impair VLDL secretion. After exclusion of these gene effects, there were consistent associations between genetically predicted NAFLD and coronary artery disease for all NAFLD traits (i.e., cALT [OR: 1.203, 95% CI: 1.113, 1.300]), imaging‐based (OR: 2.149, 95% CI: 1.276, 3.620) and biopsy‐confirmed NAFLD (OR: 1.113, 95% CI: 1.041, 1.189), and this association with coronary artery disease persisted when more stringent biopsy‐confirmed NAFLD criteria were used (OR: 1.154, 95% CI: 1.043, 1.278), or when more stringent MR methods were applied.^46^ Thus, these data emphasise that there is a robust association between genetically predicted NAFLD and coronary artery disease, after the exclusion of genetic variants that are implicated in impaired VLDL secretion.

## 5 NUTRITION, POOR DIET, LIFESTYLE CHANGE, AND TREATMENTS FOR NAFLD

There is a large body of evidence demonstrating that lifestyle change focused on weight loss, adoption of a Mediterranean diet that is low in saturated fat, and increased physical activity benefits the early stages of liver disease in NAFLD.^47^, ^48^ These studies need to be extended to patients with more advanced NAFLD and over longer periods of time, to establish if they remain tractable and effective. Indeed, the use of such diets alongside pharmacotherapies in trials remains an under‐studied area.

With the burgeoning epidemic of obesity in children, NAFLD is becoming a problem in this patient group.^49^ Certain foodstuffs that are commonly used and abused in children may also specifically increase the risk of NAFLD and promote the risk of more severe liver disease with NAFLD. One such foodstuff is sugar, and high fructose diets either in the form of refined sugar or as added corn syrup, may not only increase liver fat but may also promote liver inflammation and also increase serum uric acid levels.^50^ In 2017, in a large cohort of obese children who had undergone a liver biopsy, the late Valerio Nobili and colleagues showed that a high dietary fructose intake was independently associated with NASH and increased uric acid levels.^51^ Fructose, increases de novo lipogenesis, leads to ATP depletion and increases uric acid and increases cellular stress and inflammation.^52^ Additionally, increased fructose intake may also lead to dysbiosis (alteration of the gut microbiota) and increase gut permeability^50^; thereby increasing the potential to increase lipopolysaccharide concentrations in the portal circulation.^50^

In order to make the necessary lifestyle changes that are known to be beneficial in NAFLD, behaviour change is crucial. Unfortunately, sustained long‐term behaviour change is very difficult to achieve in an obesogenic environment and without intensive support. Moreover, few patients with NAFLD receive the necessary sustained support within modern health care systems. However, weight loss, decreased calorie intake, increased physical activity or exercise and also alcohol moderation can also result in a marked triglyceride‐lowering effect, prevent diabetes and improve cardiovascular disease risk markers.^53^, ^54^, ^55^, ^56^, ^57^, ^58^, ^59^ Specifically, in the liver, weight loss and exercise improves hepatic insulin sensitivity, decrease hepatic glucose production and decrease triglyceride accumulation^53^ and these effects would potentially be of benefit not only in NAFLD but also in T2DM.

**FIGURE 1:**
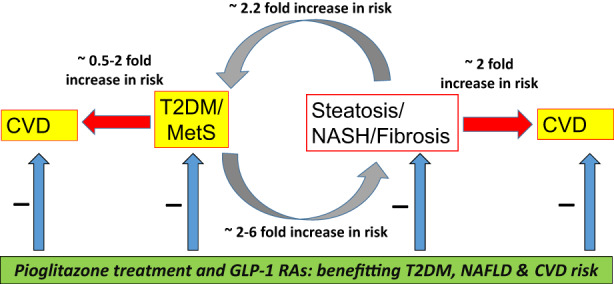
illustrates the vicious cycle that exists when NAFLD and type 2 co‐exist. NAFLD increases the risk of developing T2DM and when T2DM occurs, T2DM increases the risk of developing liver fibrosis. The figure also illustrates the relationship between both T2DM and NAFLD and the risk of developing CVD. Increasing evidence suggests that both pioglitazone and GLP‐1RAs have beneficial effects on T2DM, NAFLD, and CVD as illustrated by the negative signs in the figure. GLP‐1RAs induce weight loss, and there is also a good case for dual therapy with GLP‐1RAs and pioglitazone in order to attenuate the risk of any weight gain with pioglitazone.

Given it is likely that in the near future, there will be widespread use of glucagon‐like peptide‐1 receptor (GLP‐1R) agonists for the treatment of obesity, it is crucial that there is improved awareness of NAFLD. Health care professionals (HCPs) caring for patients with diabetes are already very familiar with this class of agents for the treatment of hyperglycaemia in T2DM. Although there are presently no licensed treatments for liver disease in NAFLD, it is important to bear in mind that NAFLD occurs very frequently with T2DM. Where T2DM is also present, clinicians should be aware that both the peroxisome proliferator‐activated receptor‐gamma agonist pioglitazone and GLP‐1RAs, are licensed for the treatment of T2DM and have proven cardiovascular benefits in people with T2DM. Since GLP‐1RAs (mostly subcutaneous liraglutide and semaglutide) and also pioglitazone, have also been shown to be of benefit for liver disease in patients with NASH,^60^ HCPs should have a low threshold for prescribing these medications (assuming there are no clinical contraindications) in patients with NAFLD who also have T2DM. Sadly, pioglitazone which is an inexpensive generic drug has become the ‘forgotten, cost‐effective and cardioprotective, drug for the treatment of T2DM’.^61^ Although pioglitazone has important well‐recognised side effects, these side effects have resulted in it not being considered a useful drug in patients with T2DM, who are at increased risk of CVD. Since pioglitazone has beneficial effects to treat hyperglycaemia, treating liver disease in NASH and also decrease CVD risk, pioglitazone should be considered in patients with T2DM who have NAFLD in whom there are no contraindications. There is evidence of efficacy to treat liver disease with both 30 and 45 mg pioglitazone doses per day and although there is limited evidence with lower doses than 30 mg/day to treat liver disease, lower doses of pioglitazone are effective at treating hyperglyceridaemia. Perhaps, therefore, there is a good case for considering a lower dose of 15 mg/day if the clinician or patient is worried about pioglitazone‐associated side effects. Since GLP‐1RAs induce weight loss, there is also a good case for combination therapy with GLP‐1RAs and pioglitazone in order to attenuate the risk of weight gain with pioglitazone. Figure 1 illustrates the vicious cycle that exists when NAFLD and type 2 co‐exist together in patients. For example, NAFLD increases the risk of developing T2DM and when T2DM occurs, T2DM increases the risk of developing liver fibrosis in patients with NAFLD. Figure 1 also illustrates the relationship between both T2DM and NAFLD, and also the risk of developing CVD. Increasing evidence suggests that both pioglitazone and GLP‐1RAs have beneficial effects on T2DM, NAFLD and CVD as illustrated by the negative signs in the figure.

A recent meta‐analysis of randomized placebo‐controlled clinical trials assessed the effect of GLP‐1RAs on the lipid profile and liver enzymes in patients with NAFLD.^62^ This analysis suggested that GLP‐1RA treatment significantly reduces liver enzymes in patients with NAFLD, but the lipid profile is unaffected.^62^ Although sodium‐glucose cotransporter‐2 (SGLT2) inhibitors‐show promise in the treatment of not only T2DM and increased risk of CVD, the evidence is equivocal that this class of drugs benefits NAFLD.^60^ In a recent systematic review^60^ that included a total of 25 active‐controlled or placebo‐controlled trials (eight for PPAR agonists, 10 for GLP‐1R agonists, and seven for SGLT2 inhibitors), 2597 individuals (1376 [53%] men vs 1221 [47%] women; mean age 52 years (SD 6); mean BMI 32 kg/m^2^ (SD 3); 1610 [62%] with T2DM) were included. Whereas this analysis showed that pioglitazone, lanifibranor, and GLP1‐R agonists (mostly liraglutide and semaglutide) improved individual histological features of NASH (i.e. steatosis, ballooning, lobular inflammation) or achieved resolution of NASH without worsening of fibrosis; the evidence was not so convincing for SGLT2 inhibitors (mostly empagliflozin and dapagliflozin). SGLT2 inhibitors reduced liver fat content, as assessed by magnetic resonance‐based techniques, but there is limited evidence of benefit to date, showing resolution of NASH or effects on liver fibrosis. Thus, the best evidence of efficacy in NAFLD is with glucose‐lowering drugs such as pioglitazone and GLP‐1RAs. Much of the effect on the liver with pioglitazone and also GLP‐1RAs, is indirect and occurs outside the liver. However, there is also evidence that pioglitazone has beneficial effects on hepatic stellate cells to potentially benefit liver disease. In contrast, there is little evidence to support a direct effect of GLP‐1RAs on liver disease in NAFLD. It seems likely therefore that most of the derived benefit of GLP‐1RAs to attenuate liver disease in NAFLD occurs via the marked GLP‐1RAs‐induced weight loss. Table 2 shows the placebo‐controlled or active‐controlled RCTs with different drugs that have PPAR gamma agonist activity for the treatment of NAFLD. These drugs include pioglitazone which is a single agonist with potent PPAR gamma activity, saroglitazar which is a dual agonist with PPAR alpha and gamma activity and lanifibranor which is a pan PPAR agonist with PPAR alpha, delta and gamma activity. Table 3 shows placebo‐controlled or active‐controlled RCTs with different GLP‐1RAs for the treatment of NAFLD or NASH.

**TABLE 2:** Placebo‐controlled or active‐controlled RCTs with different drugs that have PPAR gamma agonist activity for the treatment of NAFLD. Pioglitazone is a single agonist with PPAR gamma activity, saroglitazar is a dual agonist with PPAR alpha and gamma activity and lanifibranor is a pan PPAR agonist with PPAR alpha, delta and gamma activity)

Study	RCT characteristics	Interventions (n), RCT duration	Key efficacy outcomes	Major adverse effects
Belfort et al.^71^ USA	Adults with T2DM or prediabetes and biopsy‐confirmed NASH. Mean age: 51 years; men: 45%; BMI: 33.2 kg/m^2^; HbA1: 6.2%	A. Pioglitazone 30 mg/d for 2 months, then 45 mg/day (*n* = 29) B. Placebo (*n* = 25). Length: 24 weeks	Pioglitazone versus placebo, improvement in hepatic fat content (54% vs. 0%, *p* < 0.001), necro‐inflammation (85% vs. 38%, *p* = 0.001). Percent with liver fibrosis improvement was not significant: 46% vs. 33%, *p* = 0.08. Weight: 2.5 kg (*p* < 0.001) vs. −0.5 kg (*p* = 0.53), *p* = 0.003	Withdrawal due to AEs: 1/29 (3.5%) in pioglitazone group vs. 1/25 (4%) in the placebo group
Aithal^72^ UK	Non‐diabetic adults with biopsy‐confirmed NASH. Mean age: 53 years; men: 61%; BMI: 30.3 kg/m^2^; HbA1: NR; ALT and AST: no reported	A. Pioglitazone 30 mg/day (*n* = 37). B. Placebo (*n* = 37). Length: 52 weeks	Pioglitazone versus placebo. Number (%) with improvement (*p*‐ value), between‐groups *p*‐value: Steatosis: 15/31 (48%) (*p* = 0.001) vs. 11/30 (37%) (*p* = 0.03), *p* = 0.19. Liver fibrosis: 9/31 (29%) (*p* = 0.006) vs. 6/30 (20%) (*p* = 0.81), *p* = 0.05. Weight: 2.6 kg (*p* = 0.005) vs. −3.5 kg (*p* = 0.69), *p* = 0.02	Withdrawal due to AEs: 3/37 (8.1%) in pioglitazone group vs. 4/37 (10.8%) in the placebo group
Sanyal^73^ USA, PIVENS	Non‐diabetic adults with biopsy‐confirmed NASH. Mean age: 46 years; men: 40%; BMI: 34 kg/m^2^; ALT: 83 IU/L; AST: 56 IU/L	A. Pioglitazone 30 mg/day (*n* = 80). B. Vitamin E 800 IU/d (*n* = 84). C. Placebo (*n* = 83). Length: 96 weeks	Pioglitazone versus placebo. NASH improvement, *n* (%): 27/80 (34%) (*p* = 0.04) vs. 36/84 (43%) (*p* = 0.001) vs. 16/83 (19%). NAFLD activity score: −1.9 (*p* < 0.001) vs. ‐1.9 (*p* < 0.001) vs. −0.5. Steatosis: −0.8 (*p* < 0.001) vs. −0.7 (*p* < 0.001) vs. ‐0.1. Fibrosis: −0.4 (*p* = 0.10) vs. −0.3 (*p* = 0.19) vs. −0.1. Weight: 4.7 kg (*p* < 0.001) vs. 0.4 (*p* = 0.65) vs. 0.7 kg	Withdrawal due to AEs: None
Sharma^74^ India	Adults with biopsy‐confirmed NASH. Mean age: 39 years; men: 54%; BMI: 24.9 kg/m^2^.	A. Pentoxifylline 1200 mg/day (*n* = 29) B. Pioglitazone 30 mg/day (*n* = 30). Length: 24 weeks	Pioglitazone versus pentoxifylline. Brunt's score: −0.34 (*p* = 0.10) vs. −1.2 (*p* = 0.005), *p* = 0.04. Steatosis: −0.83 (*p* = 0.02) vs. −1.18 (*p* = 0.005), *p* = 0.60. Fibrosis: 0.08 (*p* = 0.70) vs. −0.46 (*p* = 0.19), *p* = 0.26	Withdrawal due to AEs: None
Cusi^75^ USA	Patients with T2DM or prediabetes and biopsy‐confirmed NASH. Mean age: 51 years; men: 70%; BMI: 34.4 kg/m^2^; pre‐existing T2DM: 51%	A. Pioglitazone 45 mg/day (*n* = 50). B. Placebo (*n* = 51). All patients were prescribed a hypocaloric diet. Both groups followed with an open‐label phase with pioglitazone. Length: 72 weeks (144 weeks for the open‐label phase)	Pioglitazone versus placebo. Greater than 2‐point reduction of NAS without worsening fibrosis: 29% vs. 17%, *p* < 0.001. Fibrosis; greater than 1 point improvement: 39% vs 25%, *p* > 0.05 (NS). Fibrosis mean change in score improved with pioglitazone: 0 vs. −0.5, *p* < 0.05. Pioglitazone group gained 2.5 kg, *p* < 0.05.	NR
Gawrieh^76^ USA, EVIDENCE IV	Patients with NASH or NAFLD and elevated serum ALT levels. Liver fat content was assessed by MRI‐PDFF. Mean age: 49 years; men: 53%; BMI: 34.3 kg/m^2^; pre‐existing T2DM: 52.8%	A. Saroglitazar 1 mg/day (*n* = 26) B. Saroglitazar 2 mg/day (*n* = 25) C. Saroglitazar 4 mg/day (*n* = 27) D. Placebo (*n* = 28). Length: 16 weeks	Relative changes from baseline of liver fat content at week 16 (*p*‐value) for each group: A. LS (least squares) Mean = +3.8%, SE (standard error) = 5.7, *p* = 0.97. B. LS Mean = +0.5%, SE = 6.3, *p* = 0.68. C. LS Mean = −19.7%, SE = 5.6, *p* = 0.004. D. LS Mean = +4.1%, SE = 5.9. The LS mean difference between saroglitazar and placebo (95% CI) in liver fat content at week 16 was −0.3% (95% CI ‐16.8 to 16.2) (p = 0.97), − 3.6% (95% CI ‐20.8 to 13.5) (p = 0.67), and − 23.8% (95% CI ‐39.9 to −7.7) (p = 0.004) for saroglitazar 1‐mg, 2‐mg and 4‐mg groups, respectively	AEs: 112 treatment‐ adverse events were reported in 59 patients. 13 patients in the saroglitazar 1 mg group, 13 patients in the saroglitazar 2 mg group, 14 patients in the saroglitazar 4 mg group, and 19 patients in the placebo group. No serious AEs occurred
Francque^77^ Multinational, NATIVE	Patients with biopsy‐confirmed NASH and fibrosis. Mean age: 54 years; men: 42%; BMI: 32.9 kg/m^2^; pre‐existing T2DM: 41.7%	A. Lanifibranor 800 mg/day (*n* = 83). B. Lanifibranor 1200 mg/day (*n* = 83). C. Placebo (*n* = 81). Length: 24 weeks	Lanifibranor versus placebo. Resolution of NASH with no worsening of fibrosis: 33% (*p* = 0.04 vs. placebo) in lanifibranor 800 mg group, 45% (*p* < 0.001 vs. placebo) in lanifibranor 1200 mg group, and 19% in the placebo group. Improvement in fibrosis by at least 1 stage and no worsening of NASH: 28% (*p* = 0.53 vs. placebo) in lanifibranor 800 mg group, 42% (*p* = 0.011 vs. placebo) in lanifibranor 1200 mg group, and 24% in the placebo group. Resolution of NASH and improvement of fibrosis: 21% (*p* = 0.02 vs. placebo) in lanifibranor 800 mg group, 31% (*p* < 0.001 vs. placebo) in lanifibranor 1200 mg group, and 7% in the placebo group	Serious AEs: 3 patients in the lanifibranor 800 mg group, 7 patients in the lanifibranor 1200 mg group and 3 patients in the placebo group

Abbreviations: BMI, body mass index; CI, confidence interval; MRI‐PDFF, magnetic resonance imaging‐proton density fat fraction; NAFLD, nonalcoholic fatty liver disease; NASH, nonalcoholic steatohepatitis; T2DM, type 2 diabetes mellitus.

**TABLE 3:** Placebo‐controlled or active‐controlled RCTs with different GLP‐1RAs for the treatment of NAFLD or NASH

Study	RCT characteristics	Interventions (n), RCT duration	Efficacy outcomes	Major adverse effects
Armstrong et al.^78^ UK, LEAN trial	Patients with biopsy‐confirmed NASH. Mean age: 51 years; men: 60%; BMI: 36 kg/m^2^; fibrosis F3‐F4 (on histology:) 52%; pre‐existing T2DM: 33%	A. Liraglutide 1.8 mg/day (*n* = 26). B. Placebo (*n* = 26). Duration: 48 weeks	GLP‐1RAs versus placebo. Histologic resolution of NASH: 39% vs. 9%, *p* = 0.019. Change in histologic NAS score: −1.3 vs. −0.8, *p* = 0.24. Change in fibrosis stage: −0.2 vs. 0.2, *p* = 0.11. Fibrosis improvement: 26% vs. 14%, *p* = 0.46 Fibrosis worsening: 9% vs. 36%, *p* = 0.04. Adjustment for weight loss result in non significant effect of GLP‐1RA	Gastrointestinal side effects GLP‐1RA vs. placebo: 81% vs. 65%, respectively
Dutour et al.^79^ France	Patients with T2DM, 95% of whom had NAFLD assessed by MRS. Mean age: 52 years; men: 48%; BMI: 36 kg/m^2^	A. Exenatide 5–10 mcg bd (*n* = 22). B. Placebo (*n* = 22). Duration: 26 weeks	GLP‐1RAs versus placebo. Reduction in liver fat content when compared with placebo (liver fat content: −23.8 ± 9.5% vs. +12.5 ± 9.6%, *p* = 0.007). Weight loss (−5.5 ± 1.2 kg vs. −0.2 ± 0.8 kg; *p* = 0.001 for difference between groups)	Not reported
Yan et al.^80^ China	Patients with T2DM and NAFLD were assessed by MRI‐PDFF. Mean age: 44 years; men: 69%; BMI: 29.8 kg/m^2^	A. Liraglutide 1.8 mg/day (*n* = 24). B. Insulin glargine 0.2 IU/kg/day (*n* = 24). C. Sitagliptin 100 mg/day (*n* = 27). Duration: 26 weeks	Compared to baseline. In the liraglutide and sitagliptin groups, liver fat content significantly decreased from baseline to week 26 (liraglutide: from 15.4 ± 5.6% to 12.5 ± 6.4%, *p* < 0.001; sitagliptin: from 15.5 ± 5.6% to 11.7 ± 5.0%, *p* = 0.001). Body weight was significantly decreased in the liraglutide and sitagliptin groups	Not reported
Khoo et al.^81^ Singapore	Non‐diabetic patients with obesity and NAFLD assessed by MRI‐PDFF. Mean age: 41 years; men: 90%; BMI: 33 kg/m^2^	A. Liraglutide 3.0 mg/day (*n* = 15). B. Lifestyle modifications (diet+exercise) (*n* = 15) Duration: 26 weeks	Compared to baseline. At 26 weeks, the two treatment groups showed significant (*p* < 0.01) but similar reductions in liver fat content (−8.1 ± 13.2 vs. ‐7.0 ± 7.1%).	Gastrointestinal side effects more common in the liraglutide group
Liu et al.^82^; China	Patients with T2DM and NAFLD were assessed by MRI‐PDFF. Mean age: 48 years; men: 50%; BMI: 28 kg/m^2^	A. Exenatide 1.8 mg/day (*n* = 38). B. Insulin glargine 0.2 IU/kg/day (*n* = 38). Duration: 24 weeks	Liver fat content was significantly reduced after exenatide treatment (change of liver fat: −17.6 ± 12.9%). Exenatide resulted in greater reductions in visceral adipose tissue (ΔVAT −43.6 ± 68.2 cm^2^)	Not different between groups
Bizino et al.^83^ Netherlands	Patients with T2DM and NAFLD were assessed by MRS. Mean age: 60 years; men: 59%; BMI: 32 kg/m^2^	A. Liraglutide 1.8 mg/day (*n* = 23). B. Placebo (*n* = 26) Duration: 26 weeks	Liver fat content not different between groups (liraglutide: from 18.1 ± 11.2% to 12.0 ± 7.7%; placebo: from 18.4 ± 9.4% to 14.7 ± 10.0%; estimated treatment effect −2.1% [95% CI ‐5.3 to 1.0]). Compared to placebo, liraglutide significantly reduced body weight (liraglutide: from 98.4 ± 13.8 kg to 94.3 ± 14.9 kg; placebo: from 94.5 ± 13.1 kg to 93.9 ± 3.2 kg; estimated treatment effect −4.5 kg [95% CI ‐6.4 to −2.6])	No serious drug‐related adverse events
Kuchay et al.^84^ India, D‐LIFT trial	Patients with T2DM and NAFLD were assessed by MRI‐PDFF. Mean age: 47 years; men: 70%; BMI: 29.7 kg/m^2^	A. Dulaglutide 1.5 mg/week (*n* = 32). B. Placebo (*n* = 32) Duration: 24 weeks open‐label trial (add‐on to usual care)	Dulaglutide treatment resulted in a control‐corrected absolute change in liver fat content of −3.5% (95% CI −6.6 to −0.4; *p* = 0.025) and relative change of −26.4% (95% CI ‐44.2 to −8.6; *p* = 0.004). Absolute changes in liver stiffness on Fibroscan (−1.31 kPa [−2.99 to 0.37]; *p* = 0.12) were not significant between two groups	No serious drug‐related adverse events
Guo et al.^85^ China	Patients with T2DM (treated with metformin) and NAFLD assessed by MRS. Mean age: 52 years; men: 56%; BMI: 28.7 kg/m^2^	A. Liraglutide 1.8 mg/week (*n* = 32). B. Glargine (*n* = 32). C. Placebo (*n* = 32). Duration: 26 weeks	Liraglutide treatment resulted in a control‐corrected absolute change in liver fat content of −6.3% (*p* < 0.05) and relative change of −24% (*p* < 0.05). N.B. although this change in liver fat was greater with liraglutide than with insulin glargine, there was no significant difference between the groups (−6.3 vs. −3.4%; *p* > 0.05). In the liraglutide group, there were significant decreases in body weight and waist circumference (weight, 84.3 ± 10.8 kg to 79.4 ± 9.3 kg, *p* < 0.05; and waist circumference, 95.5 ± 8.0 cm to 89.6 ± 9.3 cm, *p* < 0.05)	Mild‐to‐moderate gastrointestinal side effects were noted with liraglutide
Zhang et al.^86^ China	Patients with T2DM (treated with metformin) and NAFLD assessed by MRS. Mean age: 51 years; men: 47%; BMI: 27.3 kg/m^2^	A. Liraglutide 1.8 mg/week (*n* = 30). B. Pioglitazone 30 mg/day (*n* = 30). Duration: 24 weeks open‐label trial (add‐on to usual care)	Liraglutide treatment resulted in a control‐corrected absolute change in liver fat content of −4% (95% CI −6.6 to −0.4; *p* < 0.05) and relative change of −17% (*p* < 0.05). This change in liver fat content was greater with liraglutide than pioglitazone (e.g., 1H‐MRS (%) liraglutide baseline 24.1 ± 3.0 versus the end of study 20.1 ± 3.8; pioglitazone baseline 23.9 ± 3.8 versus the end of study 22.4 ± 3.5). In the liraglutide groups, there were significant decreases in body weight and waist circumference (weight: 79.3 ± 8.8 kg to 69.2 ± 10 kg, *p* < 0.05; and waist circumference, 93.2 ± 4.6 cm to 84.4 ± 4.0 cm, *p* < 0.05)	Mild‐to‐moderate gastrointestinal events were reported in the liraglutide group
Newsome et al.^87^ Multinational cohort of individuals from 16 countries	Patients with biopsy‐confirmed NASH and fibrosis. Mean age: 55 years; men: 41%; BMI 35.7 kg/m^2^; pre‐existing T2DM: 62%	A. Semaglutide 0.1 mg/day (*n* = 80). B. Semaglutide 0.2 mg/day (*n* = 78). C. Semaglutide 0.4 mg/day (*n* = 82). D. Placebo (*n* = 80) Length: 72 weeks	Amongst patients with stage F2 or F3 fibrosis, the percentage of patients in whom NASH resolution was achieved with no worsening of fibrosis was 40% in the 0.1‐mg group, 36% in the 0.2‐mg group, 59% in the 0.4‐mg group, and 17% in the placebo group (*p* < 0.001 for semaglutide 0.4 mg vs. placebo). Improvement in the fibrosis stage occurred in 43% of the patients in the 0.4‐mg group and in 33% of the patients in the placebo group (*p* = 0.48). Mean percent weight loss was 13% in the 0.4‐mg group and 1% in the placebo group (*p* < 0.001)	Gastrointestinal side effects were more common in the 0.4‐mg group than in the placebo group

Abbreviations: BMI, body mass index; CI, confidence interval; MRS, magnetic resonance spectroscopy; MRI‐PDFF, magnetic resonance imaging‐proton density fat fraction; NAFLD, nonalcoholic fatty liver disease; NASH, nonalcoholic steatohepatitis; T2DM, type 2 diabetes mellitus.

## 6 NAFLD IS A PUBLIC HEALTH BURDEN: WHY DOES NO COUNTRY CURRENTLY HAVE A ‘STRATEGY’ FOR NAFLD?

The economic and healthcare burden of NAFLD is considerable for all countries and especially for those countries where there is a high prevalence of obesity and T2DM. Recently, the lifetime costs of all patients with NASH in the United States in 2017 were estimated to be $222.6 billion, and amongst this group, the costs attributed to advanced NASH were $95.4 billion.^63^ However, to date despite NAFLD having a profound impact both on individual patient health and on the economics of providing health care; no countries (to date) have a dedicated strategy for providing health care to patients with NAFLD. Recently, Lazarus et al further developed and extended their European preparedness index^64^ and developed their ‘index’ to accommodate six domains with the aim of assessing how prepared countries are to deal with NAFLD.^65^ The authors assessed ‘preparedness’ by asking questions of representatives in each of these countries across six domains. These domains were (a) policy; (b) guidelines; (c) civil awareness and social engagement; (d) epidemiology and data; (e) detection; (f) care for patients with NAFLD. Responses were rated high, medium and low (according to a perceived level of ‘preparedness’); and a multiple correspondence analysis was then applied to try and assess levels of preparedness. An overall policy score for a country was then allocated, estimating ‘preparedness from a low score of 0 to a high score of 100’. A high score indicated that a country was deemed to have a high level of ‘preparedness’ for dealing with NAFLD. In this work, the authors reported the results of responses from representatives (who were mainly Hepatologists) and obtained data across 102 countries, apparently covering 86% of the global population. No country scored above half marks, and 32 countries scored zero. The findings contained in their paper led the authors to conclude that ‘although NAFLD is a pressing public health problem, no country was found to be well prepared to address it’. The authors then concluded with a call to arms stating ‘there is a pressing need for a strategy to address NAFLD at national and global levels’.

It is generally now acknowledged that NAFLD represents a public health burden that is having an impact on health care services. Moreover, there has also been a huge increase in research output and understanding in the last decades. For example, a PubMed search on the 31st December 2021, using the search term ‘NAFLD’ showed that there were 4770 citations identified by this term, compared with 22 citations in 2001. This marked increase in knowledge, and the recognition that NAFLD represents a public health burden, has resulted in many countries and economic regions establishing their own Guidelines for NAFLD over the last 6 years. The most recent of these Guidelines in December 2021 was the publication of the excellent Italian Guidelines which are a credit to the consensual working of the contributing representatives from the Italian Association for the Study of the Liver (AISF), the Italian Society of Diabetology (SID) and the Italian Society of Obesity (SIO).^66^

Nevertheless, despite the availability of Guidelines for NAFLD in many counties, there is a disconnect between the availability of guidelines, and no country has a specific NAFLD Strategy. This failing is a huge concern and such a concern should prompt us all to consider why there are no strategies for tackling NAFLD. In the opinion of the author, several key factors have impeded progress in recent years. These factors are: (a) the presence of already existing strategies for addressing obesity and T2DM, as key risk factors for NAFLD and NAFLD progression; (b) scepticism about the additional risk conveyed by NAFLD; (c) perceptions that liver fat is not harmful; (d) limited availability of non‐invasive tests for monitoring liver disease resolution or progression; (e) limited evidence of effective interventions for the amelioration of liver disease, beyond weight loss and lifestyle change; (f) the challenge of managing co‐existing multi‐morbidities such as T2DM and cardiovascular disease (CVD), which are often more urgent for patient well‐being and health; (g) the lack of licensed drug treatment for liver disease in NAFLD. In the recent work reported above,^65^ only 20 (24%) of the 83 countries that reported having guidelines for diabetes, mentioned NAFLD in these guidelines. It is important to recognise that NAFLD is very common in specialist diabetes clinics, and it is crucial that non‐specialists beyond Hepatology services, such as Diabetology and Obesity services, are involved in developing models of care for patients with NAFLD. Since there is a large gap in achieving a consensus on the model of care for patients with NAFLD is a multisystem disease, recently, a series of evidence‐based quality standard recommendations for the management of NAFLD were developed by a multidisciplinary group of experts from the British Association for the Study of the Liver and British Society of Gastroenterology NAFLD Special Interest Group, with the overall aim of improving patient care.^67^ These recommendations cover the management of people with, or at risk of, NAFLD; assessment and investigations in secondary care; management in secondary care.

**FIGURE 2:**
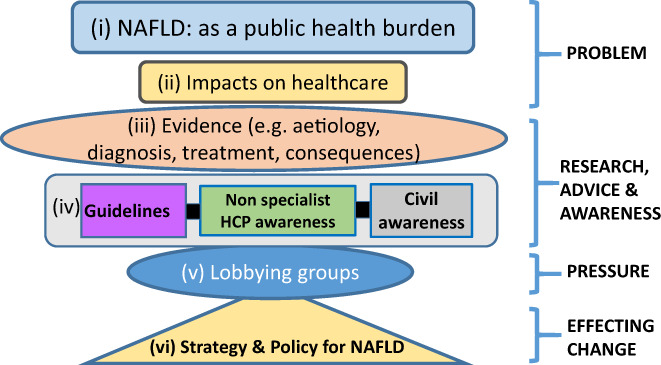
illustrates the various steps involved, (and that need to be tackled), between recognising that NAFLD is creating a problem and public health burden within society, and effecting change with the development of a health care strategy for NAFLD. (i to vi) illustrate the various steps involved in identifying NAFLD as a public health burden and establishing a strategy and policies for tackling NAFLD in society.

As has been mentioned, T2DM is a strong risk factor for liver fibrosis, cirrhosis and hepatocellular carcinoma, and glucose‐lowering drugs used in T2DM may benefit the liver in NAFLD.^68^ Since the co‐existence of T2DM and NAFLD creates a vicious spiral of worsening disease affecting both conditions,^35^ there is clearly scope for expanding diabetes guidelines to include evidence‐based information, to support NAFLD management in this patient group. A recent study based in southern and western France indicated that primary care physicians and diabetologists have limited knowledge of the chronic liver disease, despite its high prevalence.^69^ It is, therefore, crucial that there is continuing medical education amongst primary care physicians and diabetologists in order to identify those patients with more severe forms of NAFLD. Moreover, programmes focused on behaviour change, such as the English NHS Diabetes Prevention programme, afford an opportunity to extend this form of support to other groups (such as patients with NAFLD). Many patients with NAFLD may also benefit from a similar approach and benefit from lifestyle changes focused on decreasing body weight, increasing levels of physical activity, and changes to a diet where appropriate. Figure 2 illustrates the various steps involved, (and that need to be tackled), between recognising that NAFLD is creating a problem and a public health burden within society and effecting change with the development of a health care strategy for NAFLD.

## 7 CONCLUSIONS

NAFLD has become a very common condition in the 21st century. The epidemic of obesity and T2DM has had a marked impact on increasing the public health burden of NAFLD; not least because obesity is a powerful risk factor for developing NAFLD. T2DM is a strong risk factor for promoting liver fibrosis in NAFLD. NAFLD is also an independent risk factor for T2DM and CVD. Thus, the presence of co‐existing NAFLD and T2DM creates a vicious circle where NAFLD increases the risk of T2DM and the presence of T2DM increases the severity of liver disease in NAFLD. Thus, both NAFLD and T2DM could be considered ‘partners in crime’, where the presence of both ‘partners’ has a greater effect than either disease in isolation. In the last 5 years, it has become clear that NAFLD not only increases the risk of cirrhosis, primary liver cancer and end‐stage liver disease, but NAFLD is also an important multisystem disease that has major implications beyond the liver. Not only does NAFLD increase the risk of T2DM and CVD, but it has recently become clear that NAFLD is an independent risk factor for CKD and certain extra‐hepatic cancers. With the consequent health care burden created by NAFLD that has major implications for primary and secondary, it is crucial that Hepatologists work with other specialists and non‐specialists to develop strategies for NAFLD. HCPs caring for patients with diabetes are very familiar with the targeted use of pioglitazone and GLP‐1RAs in the treatment of T2DM, and these drugs have important beneficial effects on NAFLD. A paradigm change is occurring with the diabetologist/endocrinologist's greater awareness of the critical role of NAFLD in patients with T2DM^70^ and HCPs caring for patients with T2DM need to be at the heart of discussions to develop strategies for NAFLD.

## CONFLICTS OF INTEREST

The author has no competing financial interests to declare.
